# Unexplained Bilateral Simultaneous Corneal Graft Rejection in a Healthy 18-Year-Old Male

**DOI:** 10.7759/cureus.14612

**Published:** 2021-04-21

**Authors:** Hajer I Alsawad, Fatema M Aljufairi, Abdulhameed H Mahmood

**Affiliations:** 1 Ophthalmology Department, Salmaniya Medical Complex, Manama, BHR; 2 Ophthalmology Department, Prince Sultan Military Medical City, Riyadh, SAU

**Keywords:** steroid responder, corneal transplant, penetrating keratoplasty, graft rejection, cyclosporine-a, keratoconus, endothelial rejection, bilateral corneal graft rejection

## Abstract

Purpose: To report a rare case of unexplained bilateral corneal graft rejection one year after penetrating keratoplasty (PK) in an otherwise healthy individual, who was compliant with topical fluorometholone maintenance therapy.

Observations: An 18-year-old gentleman, who underwent successful, sequential, non-simultaneous, bilateral PK for advanced keratoconus, presented one year later with an acute endothelial rejection episode involving both eyes simultaneously. The rejection episode was reversed with a high dose of intravenous (IV) methylprednisolone pulse therapy over three days, topical cyclosporine-A 1%, and prednisolone acetate 1%, and then maintained on cyclosporine-A 1% eye drops, as the patient was a steroid responder.

Conclusion and importance: Bilateral corneal graft rejection, although rare, can occur even several months after successful PK. Prompt management is crucial for the successful reversal of an acute corneal graft rejection episode. In steroid responders, Cyclosporine-A 1% can play a role in reducing the need for, or frequency of, potent steroid eye drops in the acute phase, and as a long-term steroid-sparing agent for maintenance.

## Introduction

The adaptation of selective corneal tissue replacement, by anterior and posterior lamellar keratoplasty techniques over the last two decades, has revolutionized corneal graft surgery [1,2]. However, large numbers of full-thickness corneal transplants (penetrating keratoplasty [PK]) are still being performed worldwide [3-5]. Despite the advantages that deep anterior lamellar keratoplasty (DALK) provides compared to PK for young patients with keratoconus [6,7], PK is still being performed for some advanced cases of keratoconus where DALK becomes technically difficult. In a recent large retrospective study in Bahrain, keratoconus was the leading indication for PK between 1996 and 2015, accounting for 33% of all cases [8].

PK is considered one of the most successful tissue transplant procedures in medicine, with a 10-year survival rate of 65% for all patients, and as high as 95% for keratoconus patients particularly [9]. However, corneal graft rejection can have devastating effects on corneal graft survival if not recognized and treated promptly [9,10].

Aggressive topical (with or without systemic) steroid therapy remains the mainstay treatment for endothelial graft rejection [11]. In patients who are steroid responders, increased intraocular pressure can further complicate the treatment of acute endothelial graft rejection, and shifting to less potent steroid drops, or those with less propensity toward increased intraocular pressure may be necessary. Few reports even suggested the use of various concentrations of topical cyclosporine-A drops as a safe substitute to topical steroids in the treatment and prevention of corneal graft rejection, with good results [11-13].

Bilateral corneal graft rejection is a very rare presentation. In fact, only two case reports currently exist in the literature describing bilateral simultaneous corneal graft rejection [14,15]. In this paper, we report an unexplained case of bilateral simultaneous corneal graft rejection, in a healthy 18-year-old gentleman, one year after successful PK, who was also a steroid responder.

## Case presentation

An 18-year-old university student, who is a non-smoker, unknown to have any systemic illness, underwent PK for his left eye in July 2019, and his right eye in September 2019, for advanced keratoconus. His early postoperative period was unremarkable, his best-corrected visual acuity recorded in October 2019 was 6/6p in the right eye and 6/9 in the left eye, with the following refraction:

OD: -0.05/ -5.00 x 5 = 6/6p

OS: +0.50/ -6.50 x 110 = 6/9

The patient was kept on topical prednisolone acetate 1% four times a day in the early postoperative period, with anti-glaucoma drops as he was found to be a steroid responder. After slow-tapering for over six months, he was later shifted to topical fluorometholone 0.1% twice daily as maintenance therapy without any problems.

In August 2020, the patient presented with bilateral redness and a decrease in vision over the past two days. He was compliant on his topical eye drops and denied any history of eye trauma or recent illness.

**Figure 1:**
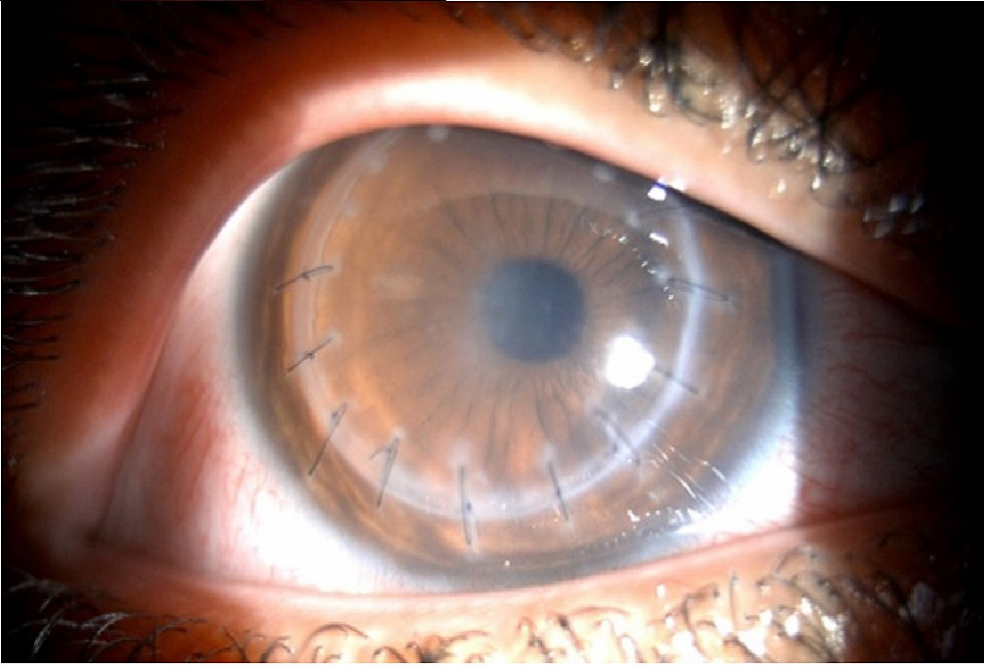
Right eye slit-lamp photo showing diffuse KPs and corneal edema. KPs: Keratic precipitates.

His best-corrected visual acuity on presentation was 6/30 in the right eye and 6/60 in the left eye. Slit-lamp biomicroscopy of the right eye (Figure 1) showed diffuse keratic precipitates (KPs) all over the corneal graft, sparing the recipient corneal rim, with moderate corneal edema. All remaining sutures were unremarkable, maintaining good tension and free of infiltration or neovascularization.

**Figure 2:**
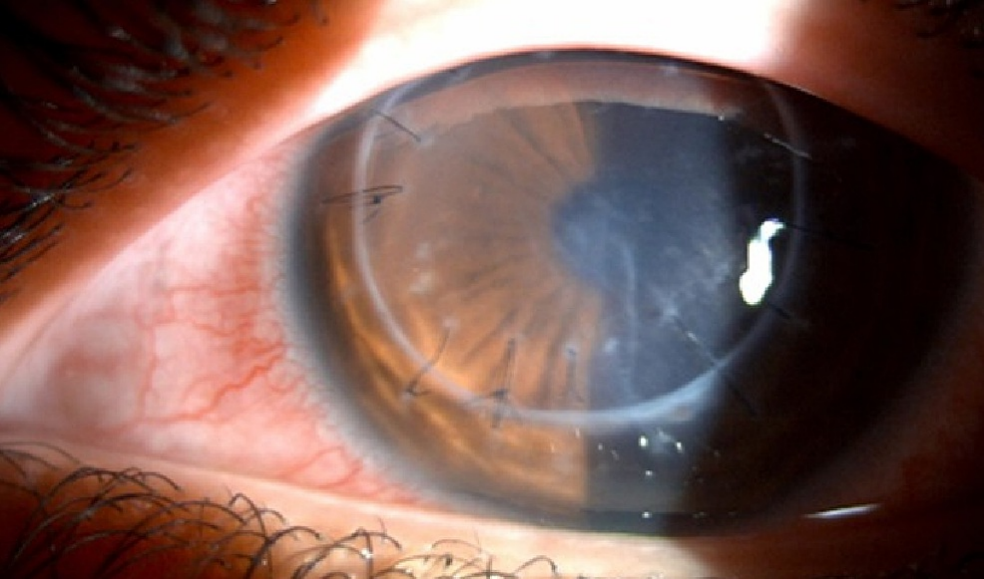
Left eye slit-lamp photo showing diffuse KPs, mild corneal edema, and central Descemet folds. KPs: Keratic precipitates.

Slit-lamp biomicroscopy of the left eye (Figure 2) showed diffuse KPs, sparing the recipient corneal rim, mild corneal edema, and few central Descemet folds. Goldmann applanation tonometry showed an intraocular pressure of 22 mmHg in the right eye and 13 mmHg in the left eye. The patient was immediately started on IV-methylprednisolone 1 g daily for three days, topical prednisolone acetate 1% every two hours, while awake, and brimonidine tartrate 0.2% three times daily.

After one week of this treatment regimen, the patient’s best-corrected visual acuity improved to 6/24 in the right eye and 6/12 in the left eye, with complete resolution of the KPs. However, mild corneal edema persisted in both eyes. The intraocular pressure at the time was 18 mmHg in the right eye and 16 mmHg in the left eye. The steroid drop was tapered four times daily and sodium chloride 5% solution was added to the regimen to improve cornea edema.

**Figure 3:**
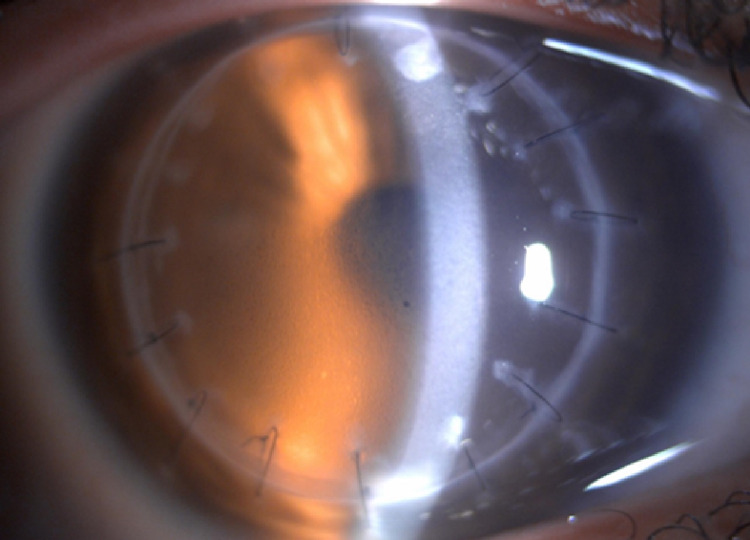
Right eye slit-lamp photo showing epithelial cystic edema due to high intraocular pressure.

**Figure 4:**
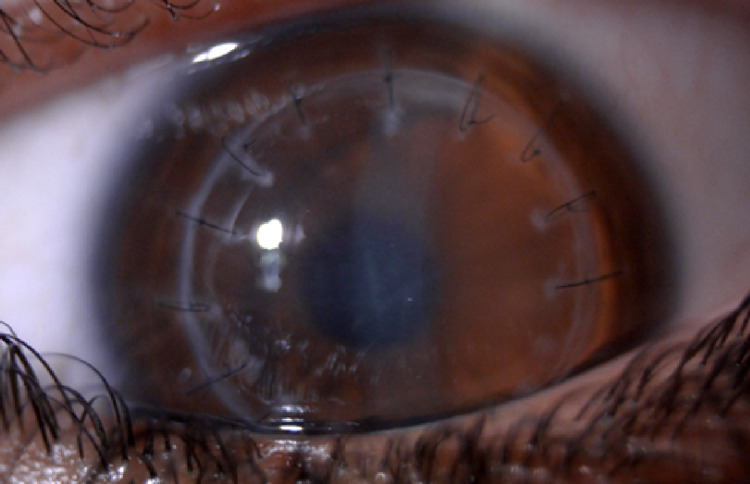
Left eye slit-lamp photo showing a clear corneal graft, after good response to treatment.

After one month, the patient presented on his follow-up appointment with the best-corrected visual acuity of 6/7.5 bilaterally. Intraocular pressure at the time was 26 mmHg in the right eye and 22 mmHg in the left eye, despite good compliance on anti-glaucoma drops (brimonidine tartrate 0.2%). Slit-lamp biomicroscopy showed epithelial cystic edema in the right eye without KPs (Figure 3), and a completely clear corneal graft in the left eye (Figure 4).

The decision was made at that point to switch the patient from prednisolone acetate 1% drops four times daily, to loteprednol etabonate 0.5% drops twice daily, in addition to cyclosporine-A 1% drops, four times daily. The patient responded well to this regimen, with intraocular pressures ranging from 12 mmHg to 16 mmHg on subsequent visits. Loteprednol etabonate drop was tapered slowly until discontinued within a month, and the patient was later maintained on fluorometholone 0.1% twice daily, in addition to cyclosporine-A 1% eye drops four times daily. The patient’s best-corrected visual acuity was last measured in December 2020, and it was 6/6 in both eyes. He’s scheduled for selective suture removal on the next follow-up appointments to address astigmatism.

## Discussion

Corneal graft rejection rarely presents in both eyes simultaneously. To the best of our knowledge, only two previous case reports exist describing simultaneous bilateral corneal graft rejection. In 1996, Solomon et al [14] reported a case of bilateral simultaneous corneal graft rejection attributed to the recent influenza vaccine. More recently, in 2020, Vanhonsebrouck et al [15] reported a case of bilateral corneal graft rejection in an 85-year-old woman, presumably associated with the initiation of pembrolizumab immunotherapy for metastatic urothelial cell tumor.

Unlike the previous two case reports, after detailed history taking, we could not find a recent event or illness that could explain the acute episode of endothelial rejection in this patient’s eyes. A deep nasal swab was negative for COVID-19, the patient had not received any vaccination prior to the episode and all blood workup came back unremarkable. The fact that the patient’s condition improved on topical steroid alone, the normal corneal sensation, the lack of iris atrophy, and the bilateral nature of the condition all practically rule out herpes simplex virus-related infection as a cause.

Prompt recognition and aggressive treatment with systemic and topical steroids proved very crucial in reversing the acute endothelial rejection episode in our patient. However, his increased intraocular pressure with the use of prednisolone acetate 1% made it necessary to switch to another steroid drop (loteprednol etabonate 0.5%) which is known to have less effect on intraocular pressure [16]. Because the decision to switch from prednisolone acetate to loteprednol etabonate was made sooner than we would usually do in cases of endothelial graft rejection, we decided to add topical cyclosporine-A 1% drops to his regimen. This addition has proven to be effective, even after discontinuation of loteprednol a month later. The patient’s grafts are currently well-maintained on weak steroid drops (fluorometholone 0.1%) and topical cyclosporine-A 1%. He is tolerating the drops well, without any local or systemic unwanted side effects, with clear corneas and best-corrected visual acuity of 6/6 bilaterally.

## Conclusions

Bilateral simultaneous corneal graft rejection is rare. Ophthalmologists should be aware of this presentation as prompt recognition and management are very crucial for the survival of the corneal graft tissue. Managing acute corneal graft rejection in a steroid responder is very challenging. After reversing an acute corneal graft rejection episode with a high-dose systemic and topical steroid, the introduction of topical cyclosporine-A 1% eye drops can be very useful in the early phase of recovery, especially in steroid responders, allowing for faster tapering off potent steroid eye drops and successfully maintaining graft clarity thereafter.
